# The different hormonal system during exercise stress coping in horses

**DOI:** 10.14202/vetworld.2020.847-859

**Published:** 2020-05-06

**Authors:** Adriana Ferlazzo, Cristina Cravana, Esterina Fazio, Pietro Medica

**Affiliations:** Department of Veterinary Sciences, Unit of Veterinary Physiology, Polo Universitario Annunziata, Messina University, 98168 Messina, Italy

**Keywords:** equines, hormones, hypothalamic-pituitary-adrenal axis, iodothyronines, physical exercise

## Abstract

The review discusses the hormonal changes during exercise stress. The exercise generally produces a rise of adrenaline (A), noradrenaline (NA), adrenocorticotropic hormone (ACTH), cortisol, glucagon, growth hormone, arginine vasopressine, etc., and a drop of insulin. The hormonal events during reestablishment of homeostasis due to exercise stress can be divided into a catabolic phase, with decreased tolerance of effort, and reversible biochemical, hormonal and immunological changes, and an anabolic phase, with a higher adaptive capacity, and enhanced performance. The two main hormonal axes activated in the catabolic phase are sympathetic–adrenal–medullary system and hypothalamic-pituitary-adrenal (HPA) axis, while in the anabolic phase, growth hormone-insulin-like factor I axis, and gonadal axes. The hormonal responses during exercise and recovery can be regarded as regulatory and integrated endocrine responses. The increase of catecholamines and ACTH is dependent on the intensity of exercise; a marked increase in plasma A occurs during exercises with high emotional content. The response of cortisol is correlated with the duration of exercise, while the effect of exercise duration on b-endorphin changes is highly dependent on the type of exercise performed. Cortisol and b-endorphin changes usually occur in phase, but not during exercises with high emotional content. Glucocorticoids and iodothyronines are involved in meeting immediate energy demands, and a model of functional interactions between HPA axis and hypothalamic-pituitary-thyroid axis during exercise stress is proposed. A modulation of coping responses to different energy demanding physical activities required for sport activities could be hypothesized. This review supports the proposed regulation of hypophysiotropic TRHergic neurons as metabolic integrators during exercise stress. Many hormonal systems (ghrelin, leptin, glucose, insulin, and cortisol) are activated to control substrate mobilizations and utilization. The cardiovascular homeostasis, the fluid and electrolyte balance during exercise are highly dependent on vasoactive hormones (antidiuretic hormone, atrial natriuretic peptide, renin–angiotensin–aldosterone, and prostaglandins) control.

## Introduction

The physical activity expressed by horses in different ways represents a disturbance to homeostasis that needs an integrative response from the entire organism. Above the need of metabolic fuel production for the working muscles, all organ systems are involved in a coordinated control to allow muscular work and adequate response to different expressions of exercise. Physical exercise enhances metabolic processes in muscle, liver, and adipose tissue to deliver energy to working muscles; it increases O_2_ consumption, respiratory, and cardiac activity and stimulates hemodynamic adjustments. The respiratory, cardiovascular, muscular, integumentary, and renal and digestive systems support the adjustments to the physical activity.

Longer work or, in sport horses, athletic performance induces system-wide alterations that require neural and endocrine mediation. The involvement of neuroendocrine system is represented not only by short-term effects, directly related to employment of exercise, but also by long-term effects, which can be the expression of an adaptation to the exercise too.

The autonomic nervous system activation is the more rapid mechanism to facilitate the rapid adjustments of the cardiopulmonary function; nevertheless, as the exercise progresses a fine-tuning of the response to exercise is necessary. It involves a multiple system response that relies on the secretion of substances produced and released by the nervous and endocrine systems, able to evoke optimal organ function and control of homeostasis of principal variables, such as mean arterial pressure, plasma osmolality, blood pH, PCO_2_, plasma electrolyte (Na^+^, K^+^, Cl^−^) concentrations, blood glucose concentration, and body temperature [1-3]. Moreover, increasing interest is devoted to the involvement of the neuroendocrine system in the psychological aspects of athletic action [4,5].

Exercise is a stress condition soliciting in the organism a new dynamic equilibrium which requires adaptive responses. The hormonal systems are involved to allow the adaptive responses to exercise. With this in mind, exercise can be considered as a useful stress model to study the interplay between the different hormonal systems in stress conditions [4,6].

Athletic performance is primarily the result of a correct integration of major body systems involved in supporting the biochemical and functional requirements of the physical activity and homeostatic adaptations. It needs also of a well-trained biomechanical activity and motivation to competition. The utilization of horses for equestrian sport activities requires preparation and optimization of specific expressions of athletic qualities, such as power, speed, endurance, jumping skill, and competitiveness. Then, in all equestrian competitions, it is necessary that horses have the peak physical fitness and the correct psychological state. Stress can potentially influence performance in horses competing in different disciplines [7,8]. Recently, the role of affective processes underpinning temperament, mood, and emotional reaction in determining discipline-specific performance has been reviewed [9]. Moreover, the use of behavioral modification techniques at assessing the emotional state of the horse during training and competition to ensure that the horse is in an appropriate psychological state for competition has been suggested [10].

In competing horses, exercise stress is determined by a mix of different factors depending on the characteristics of the exercise and on individual fitness level and perception of stress conditions. The environment and the circuit design incorporate a number of additional stressors and features to cope with. Some competitions, such as show jumping, dressage, gymkhana, and western riding, require an high degree of motor ability, shown by the capacity in the coordination of specific muscles to effect learned activities as well as in the modulation of intensity of speed.

The neuroendocrine system is directly involved in the metabolic and functional support as well as in the psychological aspects of athletic action [5,6]. The involvement of neuroendocrine system is represented by short-term effects, directly related to exercise, and by long-term effects, which can be the expression of the adaptation to exercise. Then, the neuroendocrine system plays a complex function during competitive activity as well as in training adaptations. The trophic and behavioral actions of different hormones induce tissue growth and specific adaptations, and reduce limitation to continue heavy and prolonged exercise, by conditioning the mental attitude to learning during training and the perception of effort and muscular pain during agonistic activity.

The effects of acute and chronic exercise on endocrine homeostasis have been largely documented in the horse [4,5,11-16]. Controversial data on the hormonal response to exercise depend on several modifying factors. The main modifying factors are the characteristics of the exercise (intensity, duration, type, and competitive vs. noncompetitive activity), the effect of biological rhythms, and the environmental conditions (temperature, humidity, and altitude).

It is worthwhile that endocrine variables may be useful as markers of fitness in the horse; nevertheless, correct methodological approaches to the evaluation of endocrine changes (methods of sampling, frequency, and time of sampling) should be adopted to evaluate the hormonal pattern of response to exercise.

Physical exercise stress in sport horses is considered as a physiological stress, although it can be also regarded as mental or psychogenic stressor [4,17]. As sentient beings, all animal species can show affect, emotion, feelings, and suffering, and their different cognitive ability and awareness can have a large influence on coping systems [18,19]. The response to stressors is dependent on collative factors’ influence, such as novelty, previous experience, and presence of conspecifics [20-22]. Hence, the individual response to stress is, ultimately, the function of individual “allostatic load,” i.e., the capacity of perception of stress and its control.

It is known that the changes involved in disturbing the homeostasis of an organism trigger various modifications, including an alteration in behavior, autonomic function, and overactivation of hypothalamic–pituitary–adrenal (HPA) axis [23]. Physiological stressors involve neuronal circuits that decode stimuli at the level of the brain stem; the previous experiences of mental stressors involve limbic areas, such as amygdale, hippocampus, and the frontal cortex. All stimuli converge at the paraventricular nucleus (PVN) and activate the sympathetic nervous system and the HPA axis.

Regulatory signals from the body activating the stress systems are represented by corticotrophin releasing hormone (CRH) and arginine vasopressin (AVP), as well as pro-inflammatory cytokines, tumor necrosis factor-α, interleukin 1, and interleukin 6. CRH plays a central role in the stress response by regulating the HPA axis. Experimental studies also provide evidence for the role of the endogenous opioid system in regulating and modulating the HPA axis, autonomic nervous system, and behavioral responses during stress [24,25].

Therefore, minimizing aversive stimuli and long-term stress may be beneficial not only to health and welfare but also to sport performances.

On these bases, the studies to determine the amount of acute or chronic stress on individuals during competitive or not competitive exercise stress are of increasing interest in research. Hence, the aim of this review is primarily to update the basic understanding of the endocrine responses to exercise in the horse, especially by taking into account that the neuroendocrine system, beyond its direct involvement in the context of physical effort, could have the highest significance in athletic performance, due also to psychological aspects of athletic action.

Moreover, it seems extremely significant to understand how the hormone pattern changes in response to either physical or mental components of exercise stress [5].

## The Sympathetic–adrenal–medullary (SAM) System

A major hormonal role in acute exercise is dependent on the activation of the SAM system [2,3,26,27]. The physical exercise also influences the central dopaminergic, noradrenergic, and serotoninergic systems. There is evidence in favor of changes in synthesis and metabolism of monoamines during exercise, as well as of their involvement in the control of catecholamine secretion and in the response and adaptation to exercise.

Insight into SAM activity during exercise can be obtained by direct measurement of plasma catecholamines, adrenaline (A), noradrenaline (NA), and dopamine (DA), However, methodological difficulties hamper the value of catecholamine determination for performance diagnosis of horses [1,2]. The catecholamines show high individual variability, circadian rhythm, and short half-life in blood. Within few minutes, after exercise catecholamine concentrations return to their resting levels.

Catecholamines have important roles in increasing oxygen delivery during exercise, by enhancing cardiac output, splenic erythrocyte release, and skeletal muscle blood flow. They promote muscle glycogenolysis and lipolysis in the adipose tissue, thus increasing the blood level of free fatty acids. The integrated metabolic response to catecholamines is also due to the influence on concentrations of other hormones, mainly the pancreatic hormones. Moreover, they facilitate neuromuscular transmission in skeletal muscle, stimulate contractile processes in fast twitch fibers, relax bronchioles, and stimulate respiration. During high-intensity exercise or long-duration exercise, catecholamines release increases ventilation and contributes to vasoconstriction in peripheral tissues, so increasing blood flow in cardiovascular and muscle districts [28].

Moreover, exercise training alters adrenergic receptor numbers and sensitivity in selected tissue, such as cardiac and skeletal muscles and vascular and smooth muscle [28,29].

The responses of SAM system to exercise and training in horses have been largely discussed [1,30-33]. Sympathetic nervous activity increases with intensity and duration of exercise; however, measurable changes in plasma catecholamine concentrations are not apparent below 50-70% of maximal aerobic capacity [28]. In horses during exercise, the increase in plasma NA is almost linearly proportional to exercise intensity, being higher after brief maximal exercise than after an endurance ride. On the contrary, a marked increase in plasma A occurs only during heavy exercise, especially if it is accompanied by emotional stress. The comparison of catecholamine changes after different types of exercise (sprint, multiple step exercise test, gallop, cross, and endurance) clearly showed that the increase of catecholamines is dependent on the intensity of the exercise, being the highest after the gallop. Maximal catecholamine concentration occurred just at the point of exhaustion and showed a significant correlation with blood lactate concentrations [26,27,34].

Catecholamines may be intimately involved in maximal performance. Following propranolol administration, the Thoroughbred horses exercised on a treadmill to fatigue at an intensity equivalent to 105% VO_2max_ showed a decrease in the time to fatigue. Trained horses have only minor increases of catecholamines in plasma after exercise, but basal concentrations are unchanged. Moderate-intensity short-term training reduces post-exercise increase in plasma NA, but not in plasma A.

## The HPA Axis and the Opioids

The HPA system is activated, together with the autonomic nervous system, to maintain homeostasis under stress, promoting the secretion of circulating β-endorphin, adrenocorticotropin (ACTH), and cortisol. Hypothalamic CRH and AVP, pituitary ACTH and adrenal glucocorticoids are the main components of the HPA axis. b-endorphin is an opiate-like peptide of pituitary origin derived from proopiomelanocortin, like ACTH. The HPA axis, primarily cortisol, increases hepatic glyconeogenesis and promotes lipolysis, so providing fuel for prolonged, even submaximal exercise.

In competitive horses, cortisol is considered a marker of psychophysical stress [35]. Then, to assess the exercise-induced stress the measurement of the hormones of HPA axis is usually utilized [4,5,36,37].

It is known that β-endorphin modifies the excitability of the central nervous system, inducing control of various functional mechanisms, including motor activity, and pain perception following lactic acid and catecholamine increases during exercise [35]. The rise in b-endorphin also modulates the time to fatigue and is associated to impairment of performance [15].

b-endorphin, ACTH, and cortisol changes are exclusively or partially dependent on type, intensity, and duration of exercise as well as on their interactions [27,31,38-40]. State of individual fitness and training [41,42] as well as age [43] also affect hormones’ changes; and this has been widely reported [1,2,4,5,12,35]. Catecholamines and lactate production contribute to HPA axis regulation during exercise [30]. The effects of exercise on HPA axis during competition with respect to training conditions have been also studied [44,45].

Treadmill exercises at submaximal levels produced in the horse significantly increased concentrations of b-endorphin, while ACTH concentrations did not change, but a differential response between fit and unfit horses [39]. Changes in plasma b-endorphin concentrations of horses were also recorded after 3-day event and after exercise tests on treadmill before and after acclimation to thermal stress [46].

The severity of exercise stress is associated to plasma β-endorphin changes [40]. The exercise stress individually experienced by horses affected b-endorphin changes. The previous studies reported that the effect of β-endorphin includes pain perception following changes in acid-base balance in relation to the presence of lactic acid and regulation of catecholamine secretion [15,38].

In horses, the effect of exercise duration on b-endorphin modifications was highly dependent on the type of exercise performed. During incremental exercise tests, plasma b-endorphin concentrations were positively correlated with exercise speed and intensity [39,40]. The critical threshold intensity of ≤60% VO_2max_ for significant increases in β-endorphin concentrations has been recorded [40].

Training enhances the b-endorphin response to acute exercise and the time of peak concentration differs according to the age of horses [43]. Decreased ACTH and β-endorphin concentrations were recorded after training [39], while after overtraining the mean β-endorphin concentrations of resting horses did not change [41].

Treadmill exercises at submaximal levels produced unmodified ACTH levels [39]. In contrast, acute vigorous physical exercise promptly heightened both pituitary venous blood ACTH and AVP, whose concentrations resulted correlated during incremental exercise [47]. The ACTH response resulted dependent on the intensity of exercise and showed a significant correlation with blood lactate [27] and, the recorded increase, observed during incremental exercise tests [31], was correlated with plasma AVP concentrations [47]. Moreover, novelty stimuli presented to horses during exercise tests induced plasma ACTH increase and a smaller AVP release from the posterior pituitary in addition to activating the SAM and the HPA axes and increased exercise capacity, resulting in improvement of running performance during supra-maximal exercise [47].

Cortisol secretion from the adrenal cortex is generally promoted by endogenous release following ACTH stimulation. Cortisol resting levels of sport horses appeared to be affected by stress conditions before a competition [1,2]. The increases in plasma cortisol induced by ACTH administration usually predict the exercise-induced stress response [42]. Exercises, even of mild intensity and short duration, induce a clear-cut increase in circulating cortisol concentrations [1,2]. The increases are usually correlated with the duration of exercise, although not correlated to plasma ACTH [27].

It is known that during the barrel racing and pole bending events the horse must show the best degree of motor ability. Significant increases after barrel racing and pole bending events on cortisol concentrations of quarter horses have been shown [48]. These results confirm data reported in other studies [1,2], and the previous results obtained in sport horses after exercise with the highest emotional involvement [47].

The effects of gymkhana competition on total cortisol changes were studied in Arabian pure breed horses, by taking into account the effects of the previous sport experience. Increases of cortisol concentrations after exercise were recorded, which were evident still at 30 min in inexperienced horses [49].

During competition, increases in plasma cortisol in experienced jumpers were smaller than in horses lacking experience, suggesting that horses become conditioned to the psychological stress of the show environment [1,2].

In response to exercise, cortisol and b-endorphin modifications usually occurred in phase [39,41]. However, a lack of correlation in the evolution of b-endorphin and cortisol concentrations has been reported in different stressful conditions with a high emotional content [15,46,50].

Jumping ability as well as other competitive factors (e.g., fence height) have been found to influence circulating cortisol, but not ACTH and b-endorphin concentrations; the cortisol and ACTH responses were related to the level of competition experience [37,44]. Other experimental data obtained on trained jumpers [37], performing in three competitive levels with the same circuit design over 10 fences differing for the fence height (1.10-1.30 m), showed significantly higher concentrations of β-endorphin and cortisol still 30 min after jumping. A significant effect of competitive exercise on β-endorphin and cortisol changes was recorded, while the effect of exercise on ACTH changes was exclusively seen after jumping over the highest fences. Nevertheless, the fence height and performance result only affected cortisol changes. In the course of experimental jumping exercises with different fence height (1.00-1.20 m), increases in cortisol concentrations 5 min after exercise were observed, which resulted statistically significant after performances over the highest fence heights (1.20-1.30), and when increases of β-endorphin and ACTH were also recorded. Significant positive correlations emerged between cortisol and β-endorphin and between cortisol and ACTH changes. Nevertheless, the interaction fence height/time was not statistically significant for β-endorphin, ACTH, and cortisol changes; moreover, no effect of fence height was demonstrated [37].

Negative correlations between ACTH or β-endorphin and iodothyronines’ changes in both not competitive and competitive show jumping were recorded [51].

In Standardbreds and Thoroughbreds, significant effects of both the final training sessions and competitions of 1600 m have been observed on ACTH and cortisol changes, both being superior during competition and for basal values, but exclusively in Standardbreds, as a reflex of emotional stress related to competition. In Standardbreds, the comparison between training sessions and competitive activities showed an exercise effect on β-endorphin during both training and exercise sessions [51].

## The Hypothalamic-pituitary-thyroid (HPT) Axis

The role of plasma iodothyronines in athletic performance has been investigated in different species and it is still open to debate. In horses, it needs to be further investigated [1,2,4,52-55], especially in the light that several conditions, such as stress and physical exercise, can promote changes in local iodothyronines’ metabolism, leading to different target tissue effects that depend on the presence of tissue-specific enzymatic activities [56].

In fact, it is important to underline that, besides the capacity of the thyroid gland to produce the correct iodothyronine amount, the periphery can modify the iodothyronines’ signal in time and space. Indeed, while plasma iodothyronine concentrations are relatively stable, tissues can coordinate iodothyronine concentrations through the cell-autonomous regulation of iodothyronines’ carriers, deiodinases, and receptors [57]. The intracellular availability of iodothyronines is regulated in target cells by a mechanism of activation/inactivation catalyzed by three selenoproteins: Type 1 and Type 2 iodothyronine deiodinase (D1 and D2) that converts the biologically inactive precursor thyroxine, T_4_, into T_3_, and Type 3 iodothyronine deiodinase (D3) that inactivates the iodothyronines’ action. The differential expression of deiodinases enables close control of T_3_ and its prohormone, T_4_. In humans, approximately 80% of T_3_ is produced by extrathyroidal deiodination of T_4_ mainly in liver and skeletal muscle [57]. The local regulation of iodothyronines at intracellular level enables wide fluctuations of iodothyronines in local tissues and is a powerful tool with which to modulate iodothyronine action without perturbing systemic hormone concentrations. Nevertheless, recent studies suggest that D3 plays a role in the tissue response to injury and in the balance of thyroid hormones’ (THs) homeostasis [58,59]. Moreover, the newly discovered physiological and pharmacological actions of T_4_ and T_3_ metabolites, such as 3,5-diiodothyronine (3,5-T2) and 3-iodothyronamine (T1AM), are of great interest in peripheral actions of THs [53].

THs actions can be separated into the central effects, that consist of a direct signaling on the central nervous system, and the peripheral effects that correspond to direct effects in responsive tissues. THs affect development, growth, and metabolic control, therefore being indispensable to normal development and body energy expenditure [57,60].

The main peripheral effects of THs and their metabolites are recorded in tissues, such as heart, liver, skeletal muscle, and brown adipose tissue. In target tissues, a peculiar feature of iodothyronine-dependent metabolic regulation is the acceleration of the rates of anabolic and catabolic reactions. THs act on cellular metabolism and regulate a variety of pathways that are involved in the metabolism of carbohydrates, lipids, and proteins in several target tissues. It is well known the ability of T_3_ to increase oxygen consumption in almost all cell types studied. THs also stimulate ion cycling by altering membrane permeability, the expression of ion pumps, and the characteristics of these pumps. In fact, iodothyronines are required to maintain heart rate, myocardial contractility, and vascular function [61] and influence both the type and distribution of the skeletal muscle fibers, their metabolic program and contractility apparatus [62,63].

THs have been also known to regulate energy metabolism [64,65]. T_3_ controls energy expenditure through central and peripheral pathways. T_3_ stimulates specific neurons of the ventromedial nucleus, which activate the sympathetic nervous system that in turn innervates the brown adipose tissue and leads to adaptive thermogenesis [57,60]; concomitantly, T_3_ acts directly in the brown adipose tissue and activates the thermogenic program by the control of lipid metabolism and uncoupling protein 1 activation [66].

Thyroid-stimulating hormone (TSH) and thyreoliberin (TRH) levels are also critical determinants of whole body energy metabolism. In fact, they exert thyroidal and non-thyroidal effects and thus integrate signals from nutritional status and the adrenergic nervous system with a fine regulation of iodothyronines’ production [67].

Exercise represents a condition of increased energy expenditure. In recent years, energy availability and hormonal interactions during energy availability changes in human athletes have received great attention [68]. Exercise represents also a condition of chronic stressor and THs concentrations have been considered as potentially important in the body’s adjustments to stressful conditions [59,64]. Different studies also suggest that, if exercise-related energy expenditure exceeds calories consumed, a low T_3_ syndrome may be induced [69].

Exercise can inhibit HPT and activate HPA axes [4,6]. It has been demonstrated that the increase in serum glucocorticoids and cytokines induce increase in D3 activity in liver and skeletal muscle, increasing degradation of T_3_. Moreover, the exercise has been reported to modulate a central homeostatic circuit, guaranteeing energy supply to metabolic tissues. A central role in this circuit is played by hypophysiotropic TRHergic neurons that are able to maintain adequate THs-induced lipolysis or serum glucose levels through PVN-sympathetic and parasympathetic hepatic activity [70].

It has been reported that, in response to acute or chronic stress conditions, the excitability of TRHergic neurons of the hypothalamic PVN is regulated differently through several neuromodulators [70] and the amount of exercise performed positively correlates with T_3_ and TRH mRNA [71].

Genomic and non-genomic actions of THs have been reported [56]. T_3_ primarily exerts its effects by binding to TH nuclear receptors that affect gene transcription through TH receptors (TR) in the promoter region of target genes. TR alpha and beta isoforms are encoded by two genes that are differentially expressed in various tissues. The distribution of these receptors is heterogeneous among the different tissues, and as a result some physiological effects of T_3_ are TR isoform specific. THs can also act through non-genomic mechanisms by binding to sites in the plasma membrane, such as alpha V beta 3 integrin, and the activation of cytoplasmic proteins such as AMPK, PI3K/Akt, and MAPK [56,72].

Thyroid function can be very difficult to assess in horses, especially when such assessments are attempted on the basis of determining iodothyronine concentrations in a single blood sample. This may be the reason for the lack of congruent results in horses on the practical value of blood THs evaluation for performance diagnosis and control. To better achieve this goal, TSH and TRH stimulation tests can be performed but the exogenous hormones are usually difficult to obtain.

Normal laboratory ranges of THs for sedentary horses appear to be applicable to their actively training and racing counterparts. The concentrations of THs were studied in Thoroughbred racehorses and it has been reported that the variability of plasma iodothyronines within a horse before and after exercise is remarkably high [4] that no clear-cut evidence of circadian rhythmicity of their values is recorded, that T_3_ and fT_3_ concentrations are relatively stable, while T_4_ is liable to vary considerably, and it has been also pointed out that in horses during sport activity special interpretative emphasis should be placed on T_3_ [73]. On the other hand, a plethora of non-thyroidal factors has been shown to affect the circulating level of THs in the horse [1,2,4,54].

In sport horses, different authors reported changes in basal and post-exercise THs concentrations [1,2,26,52], which often resulted dependent on different types and intensities of exercise, as extensively reported [1,2]. It has been reported that trained Thoroughbreds and Standardbreds show increased levels of T_3_ and T_4_ 1 h after swimming independent of exercise duration and a sudden increase in plasma catecholamines and T_3_ after racing [26]. It has been also reported that endurance exercise results in transient decrease in serum iodothyronine concentrations [52].

Although after prolonged submaximal exercises serum total iodothyronine concentrations remain almost unchanged, T_4_ and fT_4_ generally result significantly modified 24 h after the end of exercise. Many experimental data obtained after different types of exercise (prolonged riding-school session, light exercise session, competitive and not competitive show jumping, and standardized exercise tests) suggested a high responsiveness of free hormones to exercise and an influence of competition experience on T_3_ and fT_4_. Nevertheless, the relative workload, duration, intensity, and speed of sport activities, as well as the technical motor abilities required, can greatly modify the response of iodothyronines to exercise [1,2,4].

The studies generally showed that thyroid gland responds more slowly than the other endocrine systems. It is well known that T_3_ and free triiodothyronine (fT_3_), deriving from extra-thyroidal tissues, guarantee the supply for metabolic activity requested for the exercise.

After competitive show jumping, a significant decrease in T_3_ and fT_3_ concentrations has been recorded in trained jumpers, showing also a dependence of T_3_ and fT_3_ changes on exercise workload, results of performances and jumping ability of horses [53]. Competition experience affected T_3_ response to exercise, as demonstrated by increased concentrations of T_3_ at 15 min after a mild exercise only in non-competitors, as well as by an increase of fT_3_ levels in less experienced horses [1]. In trained horses, within physiological ranges, an increase of all iodothyronines after competition was recorded, although a significant effect of show jumping competition was recorded on fT_3_ changes [20,53]. After an experimental show jumping, T_3_ and fT_4_ changes showed statistically significant correlations to fence height. Statistically significant increases of T_3_, T_4_, and fT_4_ concentrations were recorded at 30 min. Higher than basal fT_4_ values were recorded 30 min after exercise over the highest fences [74]. A negative correlation was recorded between T_4_ and ACTH or β-endorphin changes during not competitive show jumping, while negative correlations were found between T_3_ or fT_3_ and β-endorphin changes during competition [51]. In Standardbreds, the comparison between training and competitive sessions showed an exercise effect of training exclusively on T_4_, while T_3_ concentrations significantly increased 30 min after exercise [2].

After western riding events, significant increases of total and free iodothyronine concentrations were demonstrated [48,75]. The increases were generally recorded after 30 min. However, after pole bending, an exercise characterized by maximal motor ability, the increases resulted statistically significant also at 5 min and T_3_ and T_4_ concentrations resulted still elevated after 24 h. The interaction between exercise and time resulted statistically significant exclusively for changes in T_4_ and significant positive correlations were recorded between changes in T_3_ and T_4_, fT_3_, fT_4_. Moreover, significant positive correlations between changes in cortisol and fT_3_ were found during a 6-week conventional western riding training program [75], suggesting the hypothesis of a role of THs in central arousal during sport activity.

An increase of T_3_ concentrations at 5 min post-gymkhana events were recorded exclusively in experienced horses, while no significant differences were observed for T_4_, fT_3_, and fT_4_ concentrations [49].

## Cardiovascular Homeostasis, Fluid, and Electrolyte Balance. The Vasoactive Hormones

The ability to exercise is highly dependent on an integrate control of cardiovascular and renal function. The hearth, blood vessels, and kidney play both a paracrine and endocrine role in the control of cardiovascular function [76]. The control of cardiovascular homeostasis and fluid balance is mainly produced by catecholamines. The physiological activity of A and NA, respectively, inducing vasodilation and vasoconstriction increases blood flow into skeletal muscle to enhance oxygen delivery. During exercise, the increase in blood flow to the cutaneous vascular beds also occurs as a thermoregulation-associated catecholamine effect. Cardiovascular and renal functions are also controlled by other vasoactive hormones. Exercise-induced alterations in plasma renin activity, atrial natriuretic peptide (ANP), AVP, or antidiuretic hormone (ADH), in renin–angiotensin–aldosterone system, and endothelin-1 (ET-1) in horses have been observed, as extensively reported [2,3,12,77-79]. During the onset of exercise, increased venous return results in an increase in atrial pressure and atrial stretch. Atrial stretch in turn causes a neuroendocrine response or cardiopulmonary baroreflex. The endocrine components of this cardiopulmonary baroreflex involve the release of plasma ANP by the atrial wall. ADH is secreted by the posterior pituitary gland in response to an increase in blood osmolality. ANP causes rapid vasodilation to control blood pressure elevation, urine flow, and sodium excretion. ANP also inhibits the action of other hormones, including the renin–angiotensin–aldosterone system and ADH [3]. Prostanoid actions on various portions of the cardiovascular system have received much attention. It has been demonstrated that PGE_2_ and PGI_2_ (prostacyclin) can act as vasodilators. These actions have been observed on arterioles as well as post-capillary venules. Conversely, PGF compounds have been shown to cause constriction of venules and veins. Thromboxane compounds act in platelet aggregation and can also cause vasoconstriction.

The increase in ADH concentrations is correlated with both duration and intensity of exercise [3,12]. ADH concentrations are found to increase steadily over a 60-min period of exercise. The post-exercise increase of ADH causes water retention by kidney and constriction of blood vessels, thus inducing elevation of blood pressure. After a 3-day event endurance test, ADH was elevated for 6 h. High-intensity exercise causes a reduction in renal blood flow and an elevation in plasma potassium concentration, both leading to an increase in renin release from the juxtaglomerular apparatus and a production of aldosterone from the adrenal cortex. Aldosterone reduces sodium loss and potassium excretion, so blood volume and blood pressure are increased. Incremental treadmill exercise induced an increase of plasma renin and aldosterone concentrations. Plasma renin concentrations showed only a small rise [12]. On the contrary, trotters subjected to training (run of 2000 m in track) showed a marked increase in plasma concentrations of aldosterone, being highest 1-2 h after the exercise. Maximal plasma aldosterone concentrations were found after 12 h recovery from endurance exercise and remained significantly elevated 22 h after a 3-day event endurance test. Endurance training did not affect plasma aldosterone and AVP concentrations [12].

The changes of plasma prostaglandin concentrations in horses after exercise have been investigated [11,80]. In horses exercising on a treadmill at maximum aerobic capacity, only concentrations of 6-keto-PGF_1α_, the stable metabolite of PGI_2_, were elevated above resting values, the concentration of TXB_2_ was significantly greatest at the end of post-exercise period, while PGE and PGF_2α_ concentrations were unchanged.

Erythropoietin (EPO) is a peptide hormone produced by the kidneys in response to hypoxia sensed by receptors of the renal vasculature. Recently, it has been reported that acute exercise does not induce an increase of EPO in normal horses, neither in horses performing at altitude [81]. With pronounced effects of both central and peripheral control of cardiovascular function [82,83].

Endothelins (ET-1, ET-2, and ET-3) are peptide hormones that have effects on neuroendocrine control of cardiovascular function [82,84]. Among factors affecting the release of endothelin, blood flow, ADH, and angiotensin are important [84,85]. They are potent vasoconstrictors that increase systemic arterial blood pressure and pulmonary arterial blood pressure cause alterations in cardiac output and the distribution of blood flow in the peripheral circulation [84,85]. ET-1 that has the main pronounced effect on peripheral vascular tone may control the redistribution of blood flow and blood pressure during exercise. Post-exercise increases in plasma ET-1 concentrations have been reported in horses probably due to plasma volume depletion, supporting the role of ET-1 in the modulation of vascular tone in the defense of mean arterial pressure [83,86]. An hypothetical role of ET-1 in the etiology of exercise-induced pulmonary pathologies has been supposed [87,88].

## Parathyroid Hormone (PTH)

It is widely accepted that the adrenergic system affects both calcium and PTH. PTH has been shown to have metabolic effects on the bone turnover [89,90]. Intensive exercise was associated with a marked rise in plasma PTH concentrations in young horses. No exercise-related changes in PTH were observed during prolonged low-speed exercise. Training seems to have no influence on PTH [91]. On the contrary, decreased plasma PTH levels have been detected in horses after 80 km-endurance race [92].

## Energy Balance, Anabolic, and Catabolic Hormones

Exercise results in a large metabolic challenge to the horse with an acute integrated response to fuel the bout of exertion; a long-term response is associated with the recovery from exercise. The resultant coordination of multiple physiological systems ensures adequate replenishment of stored energy and repair of tissues. Much of the integration of this response also involves the interaction of the endocrine mediators of appetite and energy balance.

Matching energy intake with energy expenditure is important for the exercising horse. Inadequate energy intake may affect elite equine athletes [93]. The mismatch in energy balance may be due to a variety of factors including disruption of the normal pattern of neuroendocrine control associated with signaling appetite and eating. Peripheral hormonal signals, such as ghrelin, adiponectin, and leptin, have been shown to play a role in mediating energy balance through their effects on feed intake, fatty acid metabolism, or glucose, and insulin regulation [94,95]. Moreover, the concentrations of these hormones may serve as indicators of energy sufficiency, providing valuable information regarding the current state of energy balance. Thus, there may be a relationship between ghrelin, leptin, and adiponectin as well as glucose, insulin, and cortisol in the physiological control of energy balance during and after exercise. In horses, peripheral ghrelin concentration is negatively correlated with body composition [94,96], while adiponectin is positively correlated [97]. In humans, adiponectin also plays a role as an insulin sensitizing agent [98]. In horses, adiponectin is negatively correlated with percent fat mass in yearling fillies and mature mares [97] and exercise has been found to increase insulin sensitivity as a result of training [99-101]. Thus, adiponectin may be an important endocrine component of this phenomenon associated with exercise-induced alterations in energy balance.

In horses, leptin is mainly secreted by white adipocytes into the peripheral blood stream in proportion to body fat percentage [97,102,103], and circulating leptin concentrations correlate with changes in energy balance in Thoroughbreds, and appear to be entrained to meal feeding in stallions [104]. With regard to exercise, data on how the type and duration of exercise affect energy balance and circulating leptin concentration in horses have not been extensively published [3].

Recent studies showed that short-term, high-intensity exercise alters plasma concentrations of active ghrelin, leptin, glucose, insulin, and cortisol in horses [95,103,105,106]. Changes in plasma post-exercise leptin and ghrelin concentrations may signal the exercised animals to alter energy intake. It has been underlined that these alterations may have functional importance that could shed light on the mechanisms behind the inappetence problems seen in some heavily exercised equine athletes [93].

Insulin is the most potent anabolic hormone, it stimulates amino acids uptake by the protein synthesis. As a consequence of the increased rate of glycogenolysis in response to exercise, a decrease in plasma insulin and an increase in glucagon concentrations can usually be recorded [11,12,107]. The decrease of insulin concentration may occur because catecholamines inhibit secretion from the pancreas. In fact, this suppression has a threshold of VO_2max_, which coincides with the increase of catecholamines [11,29]. Suppression of insulin and maintenance of blood glucose concentration have been related to the prevention of the onset of central mechanism of fatigue [108]. After exercises of different durations and intensities, the decrease in insulin concentrations has been always recorded, except for endurance exercise, where a close relationship between plasma insulin and glucose concentrations was shown. An age-related effect on the response to acute exercise both before and after 12-week of exercise training and an effect of the composition of meal ingested before exercise have been shown. Within 1 h after exercise, the concentrations of insulin increase above resting levels [107,109]. Training seems to have no effect on either the degree of suppression of plasma insulin or the time at which maximum suppression occurred, but the rebound hyperinsulinemia was less prolonged after training.

Glucagon stimulates hepatic glycogenolysis and adipose tissue lipolysis, resulting in elevated concentrations of plasma glucose, NEFAs. and glycerol, utilized by muscle and liver. Glucagon increases during exercise in the horse is necessary to maintain glucose concentration and prevents the onset of central fatigue [29,110,111]. Plasma glucagon concentrations resulted higher during a marathon race (speed=7-8 m/s) than during endurance exercise (speed=3-4 m/s) and were 20 times more those taken before exercise [110]. The increase in glucagon is altered by exercise intensity. The release of glucagon is under the influence of the increase of catecholamines [111].

During the anabolic phase, growth hormone (GH), secreted from the anterior pituitary gland, enhances protein synthesis, spares carbohydrates, and enhances fatty acids utilization. It exerts its effects through insulin-like factors or somatomedins. The physiologic role of insulin-like growth factor I (IGF-I) is mainly increasing the rate of protein synthesis [13].

The influence of exercise stress on the GH-axis can be analyzed by measurements of circulating GH, IGF-I, and their circulating binding proteins as well as measurements of GH pulsatility [112]. The physiologic role of the exercise-induced GH pulse is not fully understood. The results obtained for GH concentrations from the same sample can vary. When evaluate the measurement of IGF-I, one must consider the methodological problems associated with the binding proteins in the assays. Research is still needed to distinguish between the bound and free levels of this hormone.

GH response to acute exercise has been studied in horses [13,104,112-114]. Significant stimuli for the exercise-induced GH release have been considered to be neural input, either from muscle afferents or centrally from the motor cortex, feedback from release of IGF’s, direct stimulation by catecholamines, β-endorphin, lactate, NO, and changes in acid-base balance. As reported by McKeever [11], plasma GH concentrations show a post-exercise 8-10-fold increase. Maximal exercise can disrupt basal plasma concentrations of GH for up to 24 h of exercise. GH increase after exercise may provide an energy source during recovery by inducing lipolysis and promoting synthesis of new proteins, while, at the same time, the proteins already present in the cells are conserved. GH peak will appear within the exercise bout or immediately after exercise, and baseline values will be reached approximately 1-2 h after exercise.

Most studies indicate that GH increases in response to exercise is dependent on the relative workload and that a threshold of exercise intensity may be necessary before a significant rise in GH levels is detected [13]. GH secretory response to exercise is related to exercise intensity in a linear dose-response pattern. In equine athletes, a certain level of activity seems to be needed as well to overcome GH autonegative-feedback. After an exercise of moderate to fast trot with some cantering, the GH response of horses at 5-min was large and some horses did not respond at all, probably because the exercise stimulus was not enough to overcome autonegative-feedback of GH [113].

Some studies provide information about the acute changes in growth hormone-insulin-like factor 1 (GH-IGF-I) axis due to a bout of exercise in horses [13,104,112-114]. Nevertheless, there is no indication that exercise modifies IGF-I concentrations in plasma, neither post-race nor after an incremental treadmill exercise test [115]. The influence of factors, such as acute exercise, fitness training, time of day, sex, and age, on serum IGF-1 in normal, and healthy horses, was studied throughout a 9-wk training program. Moderate or high-intensity exercise had no effect on IGF-1 concentrations, when pre- and post-exercise values were compared [116].

One study has been performed in horses to determine the effect of exercise on 32 h GH release. The authors found a significant increase of GH 4 h after exercise. The irregular sampling intervals hinder determination of small nuances in GH pulsatility [13,112]. Hence, to induce a significant post-exercise plasma GH concentration increase, exercise of at least 10 min with intensity above lactate threshold (50-70% V0_2max_) should be performed to overcome autonegative-feedback; repeated bouts of exercise exaggerate this response.

The overall hypothesis that exercise might be a useful indicator of GH and prolactin status in horses has been studied [114]. It has been concluded that factors affecting the GH response to exercise likely preclude its usefulness as an indicator of GH status. The prolactin and GH responses to exercise differed under conditions of multiple acute exercise bout compared to extended exercise bouts, likely due to the difference in the mode of their hypothalamic regulation. It was assumed that the large variability in GH responses to exercise was due in part to the timing of exercise relative to endogenous episodes in GH and somatostatin secretion.

Sex hormones have also an anabolic effect in horses. Estrogens induce a positive effect during exercise, by promoting endurance capacity and fitness through a modulation of lipid metabolism, and by sparing muscle glycogen. Testosterone increases deposition of protein in tissues including the contractile proteins of the muscle. Basal plasma concentrations of testosterone for 26-32 h post-exercise are disrupted by exercise. In fact, an increase in plasma testosterone in response to maximal exercise was shown [41]. In Quarter horses, significant increases of 17 β-estradiol levels 30 min after training sessions and after 6 weeks of training were recorded. A significant correlation between 17β-estradiol and total iodothyronines (T_3_ and T_4_) was recorded at the end of the training period [48,75].

## Oxytocin

The oxytocin release is modulated by various stressful stimuli (fear, panic, and maximal exercises). In horses, anaerobic exercise slows the oxytocin release, but novelty stimuli associated to exercise have been reported to have no influence on plasma oxytocin concentration.

## Conclusion

As a stress, the exercise affects plasma hormone levels, generally producing a rise in concentrations of certain hormones (A, NA, ACTH, cortisol, glucagon, GH, AVP, etc.) and a drop in the concentration of other hormones (insulin, etc.). The hormonal events during reestablishment of homeostasis due to exercise stress can be divided into two phases. Initially, a catabolic phase can be distinguished, with decreased tolerance of effort, characterized by reversible biochemical, hormonal, and immunological changes. The two main hormonal axes activated in this phase are SAM and HPA axis. An anabolic phase follows, with a higher adaptive capacity and enhanced performance, in which both GH-IGF-I axis and gonadal axes are activated [117].

The hormonal changes respond to different influences. The increase of catecholamines is dependent on the intensity of the exercise, especially for NA; while a marked increase in plasma A occurs only during heavy exercise; and notably if it is accompanied by emotional stress. The intensity of exercise also influences ACTH response showing a significant correlation with blood lactate and with plasma AVP concentrations.

Cortisol and b-endorphin changes usually occur in phase in response to exercise. However, a lack of correlation in the evolution of b-endorphin and cortisol concentrations is reported in different stressful conditions with a high emotional content. The response of cortisol is correlated with the duration of exercise, while the effect of exercise duration on b-endorphin changes is highly dependent on the type of exercise performed. During incremental exercise tests plasma b-endorphin concentrations are positively correlated both with exercise speed and intensity.

Based on the data reported, the most interesting aspect of hormonal response to exercise stress seems to be played by iodothyronines. Exercise-induced changes in serum iodothyronines result to be affected by the severity of exercise, although controversial data are reported. Exercise race stress promptly induces an extra-thyroidal supply of T_3_, while endurance exertion led to a decrease. Nevertheless, usually T_4_ and fT_4_ were significantly modified 24 h after the end of exercise. A dependence of T_3_ and fT_3_ changes on some exercise-related variables (exercise workload and results of performances) and jumping ability of horses is demonstrated, thus suggesting a specific role of these hormones in supporting motivational and arousal processes during the jumping work over fences and/or metabolic needs, with a modulated contribution of thyroid and extra-thyroidal tissue production during and after exercise.

It has been reported [70] that the coping response to stress is characterized by increased synthesis and release of CRH, ACTH, and cortisol, as well as by increased TRH expression and HPT activity with iodothyronine concentrations’ increase. TRH expression depends on T_3_, which exerts an inhibitory role on the TRHergic neurons of PVN and stress history. Glucocorticoids and THs are involved in meeting immediate energy demands, thus placing the HPT and HPA axes at a central interface. Hence, a model of functional interactions between HPA and HPT axes during exercise stress, converging at PVN, could be proposed [4]. Sport activities requiring mental and motor skills could activate coping response at the level of the PVN. Mental stressors of sport activities, modulated by the previous experience or competition, could decode stimuli at the level of the brain stem and limbic areas through different neuronal catecholaminergic circuits. A modulation of coping responses to different energy-demanding physical activities required for peculiar sport activities of equines could be hypothesized. With this hypothesis in mind, this review could support the proposed regulation of hypophysiotropic TRHergic neurons as metabolic integrators during exercise stress [70].

On the other hand, the ability to perform exercise is highly dependent on efficient energy metabolism and many hormonal systems are activated to control substrate mobilizations and utilization. Short-term, high-intensity exercise also changes plasma concentrations of active ghrelin, leptin, glucose, insulin, and cortisol in horses. As a consequence of the increased rate of glycogenolysis in response to exercise, a decrease in plasma insulin and an increase in glucagon concentrations are usually recorded. The anabolic hormones (insulin, GH, and testosterone) mainly exert their effects during recovery after exercise. GH increase in response to exercise is dependent on the relative workload. Increases in serum IGF-I are reported to be transient and only observed during the onset of exercise. Significant increases in plasma testosterone and estrogens in response to exercise are shown.

The cardiovascular homeostasis, the fluid, and electrolyte balance during exercise are also highly dependent on hormonal system control. High-intensity exercise induces increases in renin release and production of aldosterone. ADH is elevated after strenuous exercise for at least 6 h. The changes in regional blood flow after exercise induce changes of plasma prostaglandin concentrations. No exercise-related changes in PTH are observed during prolonged low-speed exercise, while decreased plasma PTH levels are detected in horses after 80 km-endurance race.

## Authors’ Contributions

The idea for the paper was conceived by AF and PM. The paper was written by AF and PM. The literature search was conducted by PM, EF, and CC. All authors listed have made a substantial, direct and intellectual contribution to the work, and approved it for publication.
